# Driving and diabetes: problems, licensing restrictions and recommendations for safe driving

**DOI:** 10.1186/s40842-015-0007-3

**Published:** 2015-08-10

**Authors:** Alex J. Graveling, Brian M. Frier

**Affiliations:** 1 JJR Macleod Centre for Diabetes & Endocrinology, Aberdeen Royal Infirmary, Foresterhill, Aberdeen, AB25 2ZP UK; 2 The Queen’s Medical Research Institute, The University of Edinburgh, Edinburgh, EH16 4TJ UK

**Keywords:** Diabetes, Type 1 diabetes, Type 2 diabetes, Insulin therapy, Hypoglycemia, Automobile driving, Driving performance, Driving license, Road traffic accident, Motor vehicle accident

## Abstract

Driving is a complex process that places considerable demands on cognitive and physical functions. Many complications of diabetes can potentially impair driving performance, including those affecting vision, cognition and peripheral neural function. Hypoglycemia is a common side-effect of insulin and sulfonylurea therapy, impairing many cognitive domains necessary for safe driving performance. Driving simulator studies have demonstrated how driving performance deteriorates during hypoglycemia. Driving behavior that may predispose to hypoglycemia while driving is examined. Studies examining the risk of road traffic accidents in people with insulin-treated diabetes have produced conflicting results, but the potential risk of hypoglycemia-related road traffic accidents has led to many countries imposing restrictions on the type and duration of driving licenses that can be issued to drivers with diabetes. Guidance that promotes safe driving practice has been provided for drivers with insulin-treated diabetes, which is the group principally addressed in this review.

## Introduction

### Background

Driving has important business and recreational roles for transport in most countries, allowing people to travel to and from work, pursue their employment, and undertake multiple social and domestic activities. Most people regard driving to be a fundamental part of daily life; especially those with limited access to public transport [1]. Safe driving requires complex psychomotor skills, rapid information processing, vigilance and sound judgment [2]. Driving is classified as a light physical activity [3] but has considerable metabolic demands as has been demonstrated by driving simulator studies, which have shown high glucose consumption (predominantly cerebral) while driving [4].

Many of the microvascular and macrovascular complications of diabetes as well as some associated conditions (e.g., sleep apnea) can interfere with driving performance. Most of the cognitive functions required for driving are impaired by hypoglycemia [5, 6]. For many years diabetes, and in particular hypoglycemia, has been reported anecdotally to impair driving performance; this can lead to driving mishaps and cause road traffic accidents. Drivers have reported incidents such as driving the wrong way along motorways and injudicious parking during hypoglycemia [7].

The majority of driving licensing authorities in developed countries make a distinction between people with diabetes who require insulin therapy to treat their diabetes and those who do not. This is principally related to the risk of hypoglycemia associated with insulin therapy. Other glucose-lowering agents, particularly the insulin secretagogues, the sulfonylureas and glinides, can also cause hypoglycemia, although are seldom reviewed in relation to driving performance. Recognition that the level of accident risk depends on factors other than insulin treatment has encouraged licensing authorities to assess insulin-treated drivers individually. In some countries this has influenced changes in driving regulations to allow insulin-treated drivers who are free of complications and able to demonstrate management practises that promote driving safety (such as regular blood glucose monitoring), to be licensed to drive large commercial vehicles from which they were previously debarred. The present review focuses mainly on drivers who require insulin treatment for their diabetes.

### Literature search

A MEDLINE search (1946–2015) was conducted in January 2015 by combining the following subject terms: diabetes mellitus, diabetes mellitus type 1, diabetes mellitus type 2, automobile driving, traffic accidents, automobiles, whiplash injuries, motor vehicles and automobile driver examination. Limits of ‘human’ and ‘English language’ were imposed; the citations were then considered for relevance. Papers from the authors’ personal files were included, and lists of published references were checked to identify any other relevant material.

## Review

### Diabetes & driving performance

#### How can diabetes affect driving performance?

The considerable impact of hypoglycemia on driving performance is discussed below. Other complications of diabetes such as peripheral neuropathy, visual impairment and cerebrovascular disease leading to cognitive impairment may also affect driving performance. Peripheral vascular disease is not discussed but a lower limb amputation may impair the ability of the individual to operate the foot pedals. Adaptation of the vehicle to use hand-operated controls is a possible solution. Disorders associated with type 2 diabetes (T2DM), such as sleep apnea, can have an adverse impact on driving performance.

#### How does hypoglycemia affect driving performance?

Hypoglycemia is a common side effect of insulin therapy for diabetes for people with type 1 and type 2 diabetes [8]. Experimental laboratory studies have demonstrated that cognitive functions critical to driving (such as attention, reaction times and hand-eye coordination) are impaired during hypoglycemia [5, 9]. The changes in visual information processing that occur during hypoglycemia could affect visual perception under conditions of limited perceptual time and low visual contrast (poor light); this would also have a significant effect on driving performance [10]. Studies using a sophisticated driving simulator have shown that driving performance is affected adversely by moderate hypoglycemia, causing problems such as inappropriate speeding or braking, ignoring road signs and traffic lights and not keeping to traffic lanes [11, 12]. During simulation studies, driving *per se* required higher dextrose infusion rates to maintain normoglycemia compared to passively watching a driving video; this increased metabolic demand in drivers may risk promoting hypoglycemia, particularly if their blood glucose is <5.0 mmol/l (90 mg/dl) [4].

#### Does hyperglycemia affect driving performance?

The effect of hyperglycemia on driving performance has received very little attention and depends on how hyperglycemia is defined. A questionnaire-based study reported that hyperglycemia disrupted driving activities; 8 % of participants with T1DM reported at least one episode of disrupted driving associated with hyperglycemia over 1 year compared with 40 % of participants with insulin-treated type 2 diabetes [13]. No studies have examined the effect on driving performance but hyperglycemia does affect some measures of cognitive function and mood in people with T1DM and T2DM [14, 15].

#### How does peripheral neuropathy affect driving performance?

When reduced sensation and impaired proprioception affect the lower limbs of people with diabetes they may find it more difficult to gauge pressure on the accelerator, brake or clutch pedals. In addition many of the agents used for neuropathic pain such as gabapentin or amitriptyline can have a sedative effect, although a recent driving simulator study in people without diabetes did not show any effect of pregabalin on driving performance [16]. To our knowledge, no studies to ascertain the potential effects of peripheral neuropathy on driving performance have been performed [17].

#### How does visual impairment affect driving performance?

It is self-evident that vision is essential to safe driving performance and any impairment therefore has the potential to impair driving performance. Diabetes increases the risk of developing several eye disorders that can impair eyesight, such as cataract. Both proliferative, severe non-proliferative retinopathy and maculopathy can result in reduced visual acuity and affect visual fields. In the UK diabetes is no longer the commonest cause of blindness in working age people, but it remains a significant cause of visual impairment [18]. Because of the potential risk of visual impairment occurring through diabetic eye disease, in most westernized countries applicants for a driving license must be able to demonstrate an adequate level of vision. The assessment of people who have had photocoagulation for proliferative retinopathy usually includes the assessment of visual fields using perimetry.

#### How does cognitive impairment affect driving performance?

People with diabetes may experience cognitive impairment from various causes, including cerebrovascular disease in the older person. Despite resolution of hypoglycemic symptoms and counter regulatory hormonal responses after hypoglycemia is treated, recovery of cognitive function has been shown to lag behind the restoration of normoglycemia (between 20 to 75 min) [5, 19]. Cognitive impairment has been shown to impair driving performance both in simulator studies and in “real-world” assessments of driving performance [20].

#### How does sleep apnea affect driving performance?

Sleep apnea is associated with obesity and type 2 diabetes. With the increased prevalence of obesity in many countries people are at increased risk of developing sleep apnea. Poor sleep quality overnight leads to daytime somnolence. Performance in driving simulator studies is worse in people with sleep apnea than in those without [21, 22]. A systematic review demonstrated that the risk of road traffic accidents was three-fold higher in people with sleep apnea; treatment of sleep apnea with continuous positive airway pressure improves driving performance and reduces accident risk [23, 24].

#### Diabetes and the risk of road traffic accidents (RTAs)

This has been reviewed previously in detail for drivers with insulin-treated diabetes [25]. Some studies have reported that RTA rates appear to be no higher in drivers with diabetes [26–28] whereas other studies have reported an increased risk [29, 30]. The differences may result from the considerable heterogeneity in the design of these studies. One reason why many studies failed to show a significant difference in RTA rates at a population level between people at risk of hypoglycemia (mainly those with insulin-treated diabetes) and the general population with driving licenses is that many countries impose restrictions on drivers with insulin-treated diabetes and remove those who are at high risk of having an accident. Drivers with diabetes who have problems such as deteriorating eyesight or impaired awareness of hypoglycemia may voluntarily restrict or cease their driving activities to avoid putting themselves and others at risk. However, a RTA is likely to have multifactorial causation and it may be difficult to control for concomitant fatigue, adverse weather or road conditions, mechanical failure of the vehicle, or to the use of drugs or alcohol.

**Fig. 1:**
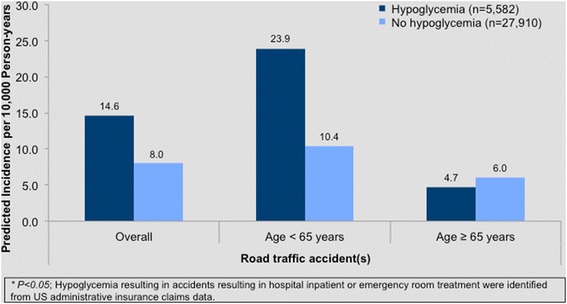
Effect of hypoglycemia requiring medical attention on incidence of road traffic accidents in people with diabetes not on insulin therapy (adapted from [33] with courtesy of Dr JE Signorovitch)

A British study suggested that drivers with insulin-treated diabetes were not at increased risk [31], but one confounding factor was that the percentages holding a driving license in populations with and without diabetes were not determined [32]. Drivers with non-insulin treated diabetes have received less scrutiny despite the fact that the risk of severe hypoglycemia with sulfonylurea therapy is similar to that of people with T2DM who have been treated with insulin for less than two years [8]. Analysis of a claims database showed that the risk of road traffic accidents was significantly increased (hazard ratio 1.8) in people with a history of requiring medical attention to treat a hypoglycemic episode; the anti-diabetes medications being used were not reported (Fig. 1) [33].

In Canada, the province of Ontario has mandatory physician reporting of drivers with medical disorders who may be unfit to drive, including people with diabetes. By providing a financial incentive for physicians to report drivers at risk (which increased the incidence of reporting) the RTA rate was lowered in those drivers who received warnings [34]. The Canadian National Population Health Survey (a large, representative longitudinal study) used self-reporting of diabetes status, insulin treatment and frequency of RTAs. The proportion of those with diabetes and/or treatment with insulin was not significantly higher in those who self-reported a history of RTAs in the preceding 12 months [35].

The largest study that examined RTA risk in drivers with diabetes analyzed data from the entire adult Norwegian population (3.1 million) for slightly over 2 years; just over 170,000 were taking anti-diabetes medications [36]. People with insulin-treated diabetes had a modestly increased risk of RTAs compared with the population as a whole, with an odds ratio of 1.4 (1.2–1.6). Those taking medication for peptic ulcers or gastro-esophageal reflux (neither of which are thought to influence driving performance) had a similar elevation in risk with an odds ratio of 1.3 (1.2–1.4). A meta-analysis of data from all these studies failed to show a significantly higher accident rate, with a non-significant risk ratio of 1.26 [37].

##### Can high-risk drivers with diabetes be identified?

Hypoglycemia is recognized to be the cause of some RTAs. When 452 people with T1DM who possessed current driving licenses were followed prospectively for 12 months with monthly reporting, 52 % reported at least one hypoglycemia-related mishap with 5 % recording as many as six or more [38]. People with a history of road traffic accidents exhibited poorer working memory during hypoglycemia compared with no history of accidents [39]. Adolescent drivers are well known to have an increased risk of accidents; parents of adolescent drivers with T1DM reported that 31 % of them had experienced a collision in the preceding year that had been attributed to hypoglycemia [40].

The factor that has most consistently been identified to be associated with an increased risk of RTAs in people with diabetes is previous exposure to severe hypoglycemia; a history of severe hypoglycemia in the preceding two years was associated with a four-fold higher risk of accidents [38, 41]. Driving problems included a variety of incidents including disruptive hypoglycemia occurring while driving and another passenger having to take over driving because of the driver’s incapacity. When adjusted for annual mileage, severe hypoglycemia, experience of hypoglycemia while driving and a previous history of a RTA within the previous two years were all associated with an increased risk of a driving mishap [38]. Stricter glycemic control (HbA1c of 7.4 % (59 mmol/mol) versus 7.9 % (63 mmol/mol)) has been reported to be associated with a higher risk of RTAs [38]. The type of diabetes was not stated in this very small study but as the mean age at diagnosis was 31.6 years and 80 % were receiving insulin therapy, most drivers presumably had T1DM [41]. However, in people with T2DM on glucose-lowering therapies, the risk of severe hypoglycemia is similar across all levels of glycemic control except in those with near-normal or very poor glycemic control, so HbA1c is unlikely to be a useful index of risk for severe hypoglycemic events and resulting RTAs [42].

Drivers with type 1 diabetes (T1DM) who had a history of driving mishaps in the previous 12 months were compared to those with those with no such history [43, 44]. Other than a greater risk of severe hypoglycemia in the later study, the groups were well matched in terms of duration of diabetes, age and glycemic control. The driving performance of those with a history of driving mishaps deteriorated to a greater extent during hypoglycemia, thus perhaps identifying a subset of drivers who are more vulnerable to hypoglycemia affecting their driving performance adversely. An 11-item questionnaire attempted to identify the “at risk” drivers with diabetes [45]. Those scoring in the upper quartile reported more driving mishaps than those in the lower quartile. The most discriminating questions regarding accident risk were those that quantified annual mileage, identified a history of hypoglycemia-related RTAs, elicited poor self-management of hypoglycemic episodes and screened for the presence of lower limb neuropathy. An internet-based management programme undertaken by drivers with T1DM reduced the frequency of driving mishaps in high-risk drivers [46].

### Hypoglycemia while driving; recognition and management

#### Experience of hypoglycemia while driving

Previous experience of hypoglycemia while driving has been reported by between 15–66 % of drivers in surveys in the United Kingdom and New Zealand [47–49]; experience of hypoglycemia while driving in the preceding year was reported by 13–29 % [26, 50]. A prospective survey in the USA observed that over a 12-month period, 41 % of drivers reported experienced “disruptive” hypoglycemia while driving; the median number of hypoglycemia episodes reported by each driver was 2.7 (range of 1–26) [38]. This would suggest that a subset of drivers experience hypoglycemia more frequently than others while driving. A small study in eastern Europe that used blinded continuous glucose monitoring showed that many drivers with T1DM develop hypoglycemia while driving, including many asymptomatic episodes [51]. In-vehicle monitoring has been suggested as a possible solution using technologies, such as continuous glucose monitoring, which are linked to the car’s dashboard display system [52].

#### The decision to drive including testing blood glucose levels before and during driving

A driver with insulin-treated diabetes needs to have accurate knowledge of their blood glucose, and appreciate the minimum level compatible with safe driving. Subjective estimates of blood glucose based on symptoms are unreliable [53–55]; this has been clearly demonstrated in drivers with T1DM before driving [47, 48]. The ability of deciding when it is safe to drive may also be unreliable or absent, particularly in those with impaired awareness of hypoglycemia. During a driving simulator study only 4 % of those with normal hypoglycemia awareness stated that they would drive while hypoglycemic compared to 43 % with impaired awareness of hypoglycemia [56]. In a laboratory study [57], 38 % of subjects felt able to drive safely when their blood glucose was 2.8 mmol/L (50 mg/dl), and in a driving simulator study three quarters of subjects neither recognized the impairment of their driving performance, nor the presence of hypoglycemia, at blood glucose levels as low as 2.8 mmol/l (50 mg/dl) [12]. It is therefore preferable that the individual’s decision to drive should be based on an actual measurement of blood glucose, though this is not enforceable in drivers with ordinary (European Group 1) driving licenses. Similar findings were observed in a prospective study when drivers with insulin-treated diabetes reported that they felt safe to drive on around 25 % of occasions when they had already ascertained that their capillary blood glucose was low (below 2.2 mmol/l (40 mg/dl)), suggesting that errors of judgment can arise from misperceptions about the safety of driving with a low blood glucose [58]. Abnormal behavior with pronounced cognitive impairment associated with hypoglycemia (becoming disorientated, getting lost or arriving at their destination with no memory of how they got there) was reported by 18 % of drivers [38], a state described in legal parlance as “automatism”.

Questionnaire-based surveys have shown that 75–91 % of drivers are able to proffer an appropriate level for the minimum blood glucose for safe driving, i.e., 4.0 mmol/l (72 mg/dl) or higher. However, it is disconcerting that almost 40–60 % of drivers with insulin-treated diabetes reported that they never test blood glucose before driving, or test only if they feel hypoglycemic [47, 50, 59]. Although testing before driving was more common in participants with impaired hypoglycemia awareness, only a small minority in this high-risk group reported regular testing. Most participants (77 %) reported never testing during journeys of any length, indicating a lack of vigilance even when the risk of hypoglycemia is not negligible [50]. This is particularly relevant in view of the metabolic demands of driving [4].

Failure to measure blood glucose could have major medico-legal consequences. In a previous prosecution within the Scottish jurisdiction, a driver with T1DM, was found guilty of causing death by dangerous driving while hypoglycemic, and was strongly criticized because he had not measured his blood glucose before driving. In passing judgment, the Sheriff highlighted the risk associated with diabetes and driving, and stated that the privilege of a driving license carries a responsibility to ensure safety by measuring blood glucose. It is important that health care professionals ensure that the potential legal consequences of not testing blood glucose in relation to driving are communicated to their patients.

#### Treatment of hypoglycemia that occurs while driving

Appropriate treatment of hypoglycemia while driving is vital. Most drivers (83–88 %) have reported that they carry carbohydrate with them in their vehicles, but very few wait for more than 30 min after self-treatment before resuming driving, although cognitive function may not have recovered fully until 45 min after restoration of normoglycemia [47, 50, 59–62]. The exact time required for recovery of sufficient cognitive function for driving has not yet been determined and drivers should be advised to err on the side of caution.

Although the definition of safe practice is debatable, any of the following omissions is unsatisfactory: not measuring blood glucose before driving; not carrying carbohydrate when driving; not stopping the vehicle when driving to self-treat hypoglycemia; and believing that a blood glucose level below 3.0 mmol/l (54 mg/dl) is compatible with safe driving. Almost half of the participants in an Edinburgh-based study failed to meet one or more of these basic standards [50]; a similar study in New Zealand reported that 33 % of drivers failed to meet one or more of these criteria [47].

### Driving regulations for drivers with insulin-treated diabetes

It is beyond the scope of this review to describe the regulations for drivers with insulin-treated diabetes in every country or continent, and in many parts of the world no such driving regulations exist. Many developed countries place restrictions on those with diabetes; the principal concern is the development of hypoglycemia while driving that may cause a RTA.

#### Driving regulations for drivers with insulin-treated diabetes in the European Union (EU)

Many European countries previously imposed a blanket ban on most drivers with insulin-treated diabetes, particularly to drive large goods vehicles or passenger carrying vehicles. This rather draconian approach failed to acknowledge that the distribution of severe hypoglycemia is skewed, with many drivers seldom or never experiencing hypoglycemia [63]. In 2006 the European Union issued its 3rd Directive on driving that addressed several medical conditions and licensing for driving, which included diabetes and aimed to harmonize the driving regulations applied by member states [64, 65]. The European regulations were also an attempt to individualize the risk associated with driving and allow some people with insulin-treated diabetes to drive Group 2 vehicles (large goods vehicles), provided they met strict criteria and could demonstrate safe driving practices.

In the EU, driving licenses are issued as a Group 1 license (an ordinary driving license for a car, light van or motorcycle) and as a Group 2 license (a vocational driving license for a large goods vehicle (LGV) or a passenger carrying vehicle (PCV)). In the United Kingdom (UK), where 40 million people hold a driving license, approximately 575,000 are held by people with diabetes, with 13 % of these being Group 2 licenses [66]. Licensing is processed by the Driver and Vehicle Licensing Agency (DVLA).

Following the issue of the 3rd EU Directive on Driving, the regulations for driving licenses have been changed throughout Europe. Medical fitness to drive has to be reviewed at least every 5 years for renewal of the driving license. In addition the requirement was introduced that any driver with diabetes holding a Group 1 driving license who experienced more than one episode of severe hypoglycemia in any 12 month period must inform their national licensing authority and the driving license revoked until this problem was addressed and the annual frequency of severe events had declined to one per year [25]. In the context of hypoglycemia risk, this change in legislation would have meant that 44 % of the intensively treated group and 17 % of the conventionally treated group in the Diabetes Control and Complications Trial (DCCT) would have lost their driving licenses at some time during the trial period [67]. A recent study in Denmark has shown that following implementation of this legislation self-reported rates of severe hypoglycemia have fallen by 55 %, suggesting that the licensing change will encourage concealment of severe hypoglycemia [68]. People with impaired awareness of hypoglycemia have also to be debarred from driving, but this condition was not defined and how this is identified and managed has been left to individual countries.

By contrast, the regulations for Group 2 licenses have been relaxed for insulin-treated drivers, who previously had been debarred from driving LGVs and PCVs. Insulin-treated drivers are now able to apply for a Group 2 license although the medical fitness requirements are stringent; they must report any severe hypoglycemic episode, must have no evidence of impaired awareness of hypoglycemia and must test their blood glucose regularly at times relevant to driving and provide an accurate diary record.

### Current guidelines for drivers with insulin-treated diabetes in the USA and Canada

The American Diabetes Association (ADA) recommends that “people with diabetes should be assessed individually, taking into account each individual’s medical history as well as the potential related risks associated with driving” [69]. With the exception of commercial interstate driving, the rules and regulations on driving and diabetes are governed by individual states and vary considerably. The Federal government in the United States does not impose any specific restrictions regarding driving for people with diabetes who are not treated with insulin. Similar to European regulations, drivers with insulin-treated diabetes may be able to obtain a driving license for commercial vehicles such as large trucks, but may not be able to cross state boundaries. Canada also imposes restrictions on driving licenses that are similar to Europe and the USA [70].

### Driving regulations in other countries

Many developing countries, such as most in sub-Saharan Africa, place no restriction on drivers with diabetes, and surprisingly these still do not exist in most advanced countries in the Middle East. The lack of driving regulations in general, and the absence of restrictions for medical disorders that can affect driving, are reflected by the high mortality and accident rate associated with road traffic accidents in these countries.

The Australian National Transport Commission have issued guidelines that promote safe driving for people with diabetes [71]. Drivers who are not insulin-treated are issued with an unconditional driving license, provided certain criteria are met (including co-morbidities). People requiring insulin treatment are issued with a period-restricted (time limited) driving license, which includes professional drivers with the equivalent of Group 2 driving licenses. In contrast with the EU, if a person with diabetes in Australia experiences severe hypoglycemia they must cease driving for a minimum period of six weeks. In addition, any driver who has a persistent loss of hypoglycemia awareness is considered unfit to drive unless their ability to experience early warning symptoms is restored.

Recommendations for safe driving practice have been issued for drivers with diabetes, an example of these is shown in Table 1.

**Table 1:** Recommendations for safe driving practice for drivers with insulin-treated diabetes [25, 72]

• Always carry your glucose meter and blood glucose strips with you
• Check your blood glucose no more than 1 h before the start of the first journey and every two hours whilst you are driving
• If driving multiple short journeys, it is not necessary to test before each additional journey as long as you test every 2 h while driving. More frequent testing may be required in circumstances where a greater risk of hypoglycemia is present, e.g., after physical activity or altered meal routine
• Try to ensure that blood glucose is kept above 5.0 mmol/l (90 mg/dl) while driving. If your blood glucose is 5.0 mmol/l or less, have a snack. Do not drive if blood glucose is less than 4.0 mmol/l (72 mg/dl) or you feel hypoglycemic
• If hypoglycemia develops while driving, stop the vehicle in a safe location as soon as possible
• Always keep an emergency supply of fast-acting carbohydrate such as glucose tablets or sweets within easy reach inside the vehicle
• Do not start driving until 45 min after blood glucose has returned to normal (confirmed by measuring blood glucose). It takes time for the brain to recover fully from hypoglycemia
• Carry personal identification to indicate that you have diabetes in case of injury
• Particular care should be taken during changes of insulin regimen, change in lifestyle, following exercise, during travel and during pregnancy
• Take regular meals, snacks and periods of rest on longer journeys. Do not drink alcohol before, or while driving

## Conclusions

Driving is a complex activity that is both mentally and physically demanding; drivers often underestimate these demands. Diabetes can impair driving performance in several ways, through short-term metabolic and longer-term complications. Hypoglycemia is a common side effect of insulin or sulfonylurea therapy and may occur during driving. Driving simulator studies have shown a decline in driving performance and impaired judgment during hypoglycemia. Despite the risks associated with hypoglycemia and driving, several surveys have shown that drivers with insulin-treated diabetes continue to embrace unsafe practices.

Many developed countries have instituted restrictions on drivers with diabetes through statutory regulations that limit the duration and scope of driving licenses. Recommendations and guidance for drivers with insulin-treated diabetes and their medical attendants have been developed, but such advice is absent in many parts of the world, where driving regulations are either very limited or non-existent. This remains a serious challenge to road and public safety in these countries and a risk to all road users.

Although the magnitude of the effects of hypoglycemia while driving on accident risk continues to be debated, hypoglycemia undoubtedly does cause road traffic accidents, some of which have a fatal outcome. Patients prone to debilitating hypoglycemia (such as those with impaired awareness of hypoglycemia) should therefore merit special consideration from the licensing authorities. The adoption of a more individualized approach to the assessment of the medical fitness to drive in North America and in Europe has been an enlightened and commendable development in recent years, but still requires further refinement to ensure its safe and effective application.

## References

[1] Sherman FT (2006). Driving: the ultimate IADL. Geriatrics.

[2] Frier BM, Frier BM, Heller SR, McCrimmon RJ (2014). Living with hypoglycaemia. Hypoglycaemia in Clinical Diabetes.

[3] Ainsworth BE, Haskell WL, Herrmann SD, Meckes N, Bassett DR, Tudor-Locke C (2011). Compendium of Physical Activities: a second update of codes and MET values. Med Sci Sports Exerc.

[4] Cox DJ, Gonder-Frederick LA, Kovatchev BP, Clarke WL (2002). The metabolic demands of driving for drivers with type 1 diabetes mellitus. Diabetes Metab Res Rev.

[5] Warren RE, Frier BM (2005). Hypoglycaemia and cognitive function. Diabetes Obes Metab.

[6] Graveling AJ, Deary IJ, Frier BM (2013). Acute hypoglycemia impairs executive cognitive function in adults with and without type 1 diabetes. Diabetes Care.

[7] Frier BM, Matthews DM, Steel JM, Duncan LJ (1980). Driving and insulin-dependent diabetes. Lancet.

[8] UK Hypoglycaemia Study Group (2007). Risk of hypoglycaemia in types 1 and 2 diabetes: effects of treatment modalities and their duration. Diabetologia.

[9] Inkster B, Frier BM (2012). The effects of acute hypoglycaemia on cognitive function in type 1 diabetes. Br J Diabetes Vasc Dis.

[10] McCrimmon RJ, Deary IJ, Huntly BJ, MacLeod KJ, Frier BM (1996). Visual information processing during controlled hypoglycaemia in humans. Brain.

[11] Cox DJ, Gonder-Frederick L, Clarke W (1993). Driving decrements in type I diabetes during moderate hypoglycemia. Diabetes.

[12] Cox D, Gonder-Frederick L, Kovatchev B, Julian D, Clarke W (2000). Progressive hypoglycemia’s impact on driving simulation performance. Occurrence, awareness and correction. Diabetes Care.

[13] Cox DJ, Ford D, Ritterband L, Singh H, Gonder-Frederick L (2011). Disruptive effects of hyperglycemia on driving in adults with type 1 and type 2 diabetes. Diabetes Care.

[14] Sommerfield AJ, Deary IJ, Frier BM (2004). Acute hyperglycemia alters mood state and impairs cognitive performance in people with type 2 diabetes. Diabetes Care.

[15] Cox DJ, Kovatchev BP, Gonder-Frederick LA, Summers KH, McCall A, Grimm KJ (2005). Relationships between hyperglycemia and cognitive performance among adults with type 1 and type 2 diabetes. Diabetes Care.

[16] Tujii T, Kyaw WT, Iwaki H, Nishikawa N, Nagai M, Kubo M (2014). Evaluation of the effect of pregabalin on simulated driving ability using a driving simulator in healthy male volunteers. Int J Gen Med.

[17] Yale SH, Hansotia P, Knapp D, Ehrfurth J (2003). Neurologic conditions: assessing medical fitness to drive. Clin Med Res.

[18] Liew G, Michaelides M, Bunce C (2014). A comparison of the causes of blindness certifications in England and Wales in working age adults (16–64 years), 1999–2000 with 2009–2010. BMJ Open.

[19] Zammitt NN, Warren RE, Deary IJ, Frier BM (2008). Delayed recovery of cognitive function following hypoglycemia in adults with type 1 diabetes: effect of impaired awareness of hypoglycemia. Diabetes.

[20] Carr DB, Ott BR (2010). The older adult driver with cognitive impairment: “It’s a very frustrating life”. JAMA.

[21] George CF, Boudreau AC, Smiley A (1996). Simulated driving performance in patients with obstructive sleep apnea. Am J Respir Crit Care Med.

[22] Stradling J (2008). Driving and obstructive sleep apnoea. Thorax.

[23] Orth M, Duchna HW, Leidag M, Widdig W, Rasche K, Bauer TT (2005). Driving simulator and neuropsychological [corrected] testing in OSAS before and under CPAP therapy. Eur Respir J.

[24] Ellen RL, Marshall SC, Palayew M, Molnar FJ, Wilson KG, Man-Son-Hing M (2006). Systematic review of motor vehicle crash risk in persons with sleep apnea. J Clin Sleep Med.

[25] Inkster B, Frier BM (2013). Diabetes and driving. Diabetes Obes Metab.

[26] Stevens AB, Roberts M, McKane R, Atkinson AB, Bell PM, Hayes JR (1989). Motor vehicle driving among diabetics taking insulin and non-diabetics. BMJ.

[27] Songer TJ, LaPorte RE, Dorman JS, Orchard TJ, Cruickshanks KJ, Becker DJ (1988). Motor vehicle accidents and IDDM. Diabetes Care.

[28] Cox DJ, Kovatchev B, Vandecar K, Gonder-Frederick L, Ritterband L, Clarke W (2006). Hypoglycemia preceding fatal car collisions. Diabetes Care.

[29] Songer TJ, Dorsey RR (2006). High risk characteristics for motor vehicle crashes in persons with diabetes by age. Annu Proc Assoc Adv Automot Med.

[30] Cox DJ, Penberthy JK, Zrebiec J, Weinger K, Aikens J, Frier B (2003). Diabetes and driving mishaps: frequency and correlations from a multinational survey. Diabetes Care.

[31] Lonnen KF, Powell RJ, Taylor D, Shore AC, MacLeod KM (2008). Road traffic accidents and diabetes: insulin use does not determine risk. Diabet Med.

[32] Major HG, Rees SD, Frier BM (2009). Driving and diabetes: DVLA response to Lonnen et al. Diabet Med.

[33] Signorovitch JE, Macaulay D, Diener M, Yan Y, Wu EQ, Gruenberger JB (2013). Hypoglycaemia and accident risk in people with type 2 diabetes mellitus treated with non-insulin antidiabetes drugs. Diabetes Obes Metab.

[34] Redelmeier DA, Yarnell CJ, Thiruchelvam D, Tibshirani RJ (2012). Physicians’ warnings for unfit drivers and the risk of trauma from road crashes. N Engl J Med.

[35] Vingilis E, Wilk P (2012). Medical conditions, medication use, and their relationship with subsequent motor vehicle injuries: examination of the Canadian National Population Health Survey. Traffic Inj Prev.

[36] Skurtveit S, Strom H, Skrivarhaug T, Morland J, Bramness JG, Engeland A (2009). Road traffic accident risk in patients with diabetes mellitus receiving blood glucose-lowering drugs. Prospective follow-up study. Diabet Med.

[37] Bieber-Tregear M, Funmilayo D, Amana A, Connor D, Tregear S (2011). Diabetes and commercial motor vehicle safety. Department of Transportation’s Federal Motor Carrier Safety Administration.

[38] Cox DJ, Ford D, Gonder-Frederick L, Clarke W, Mazze R, Weinger K (2009). Driving mishaps among individuals with type 1 diabetes: a prospective study. Diabetes Care.

[39] Campbell LK, Gonder-Frederick LA, Broshek DK, Kovatchev BP, Anderson S, Clarke WL (2010). Neurocognitive Differences Between Drivers with Type 1 Diabetes with and without a Recent History of Recurrent Driving Mishaps. Int J Diabetes Mellitus.

[40] Cox DJ, Gonder-Frederick LA, Shepard JA, Campbell LK, Vajda KA (2012). Driving safety: concerns and experiences of parents of adolescent drivers with type 1 diabetes. Pediatr Diabetes.

[41] Redelmeier DA, Kenshole AB, Ray JG (2009). Motor vehicle crashes in diabetic patients with tight glycemic control: a population-based case control analysis. PLoS Med.

[42] Lipska KJ, Warton EM, Huang ES, Moffet HH, Inzucchi SE, Krumholz HM (2013). HbA1c and risk of severe hypoglycemia in type 2 diabetes: the Diabetes and Aging Study. Diabetes Care.

[43] Cox DJ, Kovatchev BP, Anderson SM, Clarke WL, Gonder-Frederick LA (2010). Type 1 diabetic drivers with and without a history of recurrent hypoglycemia-related driving mishaps: physiological and performance differences during euglycemia and the induction of hypoglycemia. Diabetes Care.

[44] Cox DJ, Kovatchev BP, Gonder-Frederick LA, Clarke WL (2003). Physiological and performance differences between drivers with type 1 diabetes with and without a recent history of driving mishaps: a exploratory study. Can J Diabetes.

[45] Cox DJ, Singh H, Lorber D (2013). Diabetes and driving safety: science, ethics, legality and practice. Am J Med Sci.

[46] Cox DJ, Gonder-Frederick L, Ritterband L, Clarke WL, Kovatchev BP, Schmidt K (2014). Internet intervention designed to identify and reduce risk of diabetic driving mishaps (260-OR). Diabetes.

[47] Bell D, Huddart A, Krebs J (2010). Driving and insulin-treated diabetes: comparing practices in Scotland and New Zealand. Diabet Med.

[48] Arffa S (2007). The relationship of intelligence to executive function and non-executive function measures in a sample of average, above average, and gifted youth. Arch Clin Neuropsychol.

[49] Eadington DW, Frier BM (1989). Type 1 diabetes and driving experience: an eight-year cohort study. Diabet Med.

[50] Graveling AJ, Warren RE, Frier BM (2004). Hypoglycaemia and driving in people with insulin-treated diabetes: adherence to recommendations for avoidance. Diabet Med.

[51] Broz J, Donicova V, Brabec M, Janickova Zdarska D, Polak J (2013). Could continuous glucose monitoring facilitate identifying diabetes patients with a higher risk of hypoglycemia during driving?. J Diabetes Sci Technol.

[52] Kerr D, Olateju T (2010). Driving with diabetes in the future: In-vehicle medical monitoring. J Diabetes Sci Technol.

[53] Pramming S, Thorsteinsson B, Bendtson I, Binder C (1990). The relationship between symptomatic and biochemical hypoglycaemia in insulin-dependent diabetic patients. J Intern Med.

[54] Weinger K, Jacobson AM, Draelos MT, Finkelstein DM, Simonson DC (1995). Blood glucose estimation and symptoms during hyperglycemia and hypoglycemia in patients with insulin-dependent diabetes mellitus. Am J Med.

[55] Cox DJ, Clarke WL, Gonder-Frederick L, Pohl S, Hoover C, Snyder A (1985). Accuracy of perceiving blood glucose in IDDM. Diabetes Care.

[56] Stork AD, van Haeften TW, Veneman TF (2007). The decision not to drive during hypoglycemia in patients with type 1 and type 2 diabetes according to hypoglycemia awareness. Diabetes Care.

[57] Weinger K, Kinsley BT, Levy CJ, Bajaj M, Simonson DC, Cox DJ (1999). The perception of safe driving ability during hypoglycemia in patients with type 1 diabetes mellitus. Am J Med.

[58] Clarke WL, Cox DJ, Gonder-Frederick LA, Kovatchev B (1999). Hypoglycemia and the decision to drive a motor vehicle by persons with diabetes. JAMA.

[59] Watson WA, Currie T, Lemon JS, Gold AE (2007). Driving and insulin-treated diabetes: who knows the rules and recommendations?. Practical Diabetes.

[60] Evans ML, Pernet A, Lomas J, Jones J, Amiel SA (2000). Delay in onset of awareness of acute hypoglycemia and of restoration of cognitive performance during recovery. Diabetes Care.

[61] Blackman JD, Towle VL, Lewis GF, Spire JP, Polonsky KS (1990). Hypoglycemic thresholds for cognitive dysfunction in humans. Diabetes.

[62] Gonder-Frederick LA, Cox DJ, Driesen NR, Ryan CM, Clarke WL (1994). Individual differences in neurobehavioral disruption during mild and moderate hypoglycemia in adults with IDDM. Diabetes.

[63] Pedersen-Bjergaard U, Pramming S, Heller S, Wallace T, Rasmussen A, Jørgensen H (2004). Severe hypoglycaemia in 1076 adult patients with type 1 diabetes: influence of risk markers and selection. Diabetes Metab Res Rev.

[64] The European Parliament and the Council of the European Union (2006). Directive 2006/126/EC of the European Parliament and of the council of 20 December 2006 on driving licences. Off J Eur Union.

[65] The European Parliament and the Council of the European Union (2009). Commission Directive 2009/113/EC of 25 August 2009 amending Directive 2006/126/EC of the European Parliament and of the Council on driving licences. Off J Eur Union.

[66] Parkes A, Tong S, Fernandez-Medina K (2014). The forgotten risk of driving with hypoglycaemia in type 2 diabetes. Transport Research Laboratory.

[67] Kilpatrick ES, Rigby AS, Warren RE, Atkin SL (2013). Implications of new European Union driving regulations on patients with Type 1 diabetes who participated in the Diabetes Control and Complications Trial. Diabet Med.

[68] Akram K, Pedersen-Bjergaard U, Borch-Johnsen K, Thorsteinsson B (2006). Frequency and risk factors of severe hypoglycemia in insulin-treated type 2 diabetes: a literature survey. J Diabetes Complications.

[69] American Diabetes A, Lorber D, Anderson J, Arent S, Cox DJ, Frier BM (2014). Diabetes and driving. Diabetes Care.

[70] Begg IS, Yale JF, Houlden RL, Rowe RC, McSherry J (2003). Candian Diabetes Association’s Clinical Practice Guidelines for Diabetes and Private and Commercial Driving. Can J Diabetes.

[71] Austroads (2012). Assessing fitness to drive for commercial and private vehicle drivers.

[72] DVLA (2014). At a glance guide to the current medical standards of fitness to drive.

